# Impact of body mass index on long-term mortality after coronary artery bypass grafting: a retrospective cohort study

**DOI:** 10.1097/MS9.0000000000003432

**Published:** 2025-05-30

**Authors:** Jarosław Bis, Paulina Kania-Olejnik, Kamil Padaż, Marcin Malinowski, Marek A. Deja

**Affiliations:** a Department of Cardiac Surgery, Upper-Silesian Heart Center, Katowice, Poland; b Department of Cardiac Surgery, Medical University of Silesia, Katowice, Poland; c Department of Surgery, Upper-Silesian Heart Center, Katowice, Poland

**Keywords:** body mass index, coronary artery bypass grafting, mortality, obesity paradox

## Abstract

**Objectives:**

The obesity paradox in cardiac surgery suggests that obesity may be protective and associated with better survival after surgery. The aim of this study was to assess the impact of patients’ body mass index (BMI) on late mortality after isolated coronary artery bypass grafting (CABG).

**Methods:**

All consecutive patients who underwent isolated CABG at our institution from 2014 to 2020 were included. Patients were divided into four groups according to (BMI): underweight (BMI <20.0 kg/m^2^), normal weight (BMI 20.0–24.9 kg/m^2^), overweight (BMI, 25–30 kg/m^2^), and obese (BMI > 30 kg/m^2^). The long-term mortality was analyzed as primary end-point. The univariable and multivariable analysis was performed using Cox regression modeling.

**Results:**

The study population consisted of 6448 patients including 104 (1.6%), 1296 (20.1%), 2946 (45.7%), and 2102 (32.6%) in the consecutive study groups. Mean follow-up time was 4.69 ± 2.17 years. Overall 30-day mortality was 2.5%. Univariable analysis showed survival benefit in all patients with increased weight in comparison to normal weight group; with HR 0.776, 95% CI 0.675–0.891, *P* < 0.001 in the overweight, and HR 0.767, 95% CI 0.661–0.890, *P* < 0.001 in the obese patients group. Multivariable analysis revealed better survival in both overweight and obese patients in comparison to normal weight group (HR 0.836, 95% CI 0.727–0.961, *P* = 0.012, and HR 0.854, 95% CI 0.734–0.994, *P* = 0.042, respectively).

**Conclusions:**

Increased weight is associated with better long-term survival in patients after CABG, both in the overweight and obese patients

## Introduction

Obesity is a key factor in the development of several health disorders. It affects all ages and both sexes. Epidemiologists estimate that this condition has doubled since 1980 and now more than a third of the world population is classified as overweight or obese[1]. Obesity is a risk factor for cardiovascular disease including atherosclerosis, diabetes, hypertension, heart failure, and atrial fibrillation^[2,3]^. Nevertheless, in patients with established symptomatic coronary artery disease (CAD) increased body mass index (BMI) and other parameters of body composition are not consistently linked with adverse outcomes of CAD^[4,5]^. This condition is called “obesity paradox” and has also been identified in such diseases as atrial fibrillation and heart failure[6].

**HIGHLIGHTS**

- Obesity is suggested to have beneficial impact on survival in patients undergoing coronary artery bypass grafting (CABG).
- This phenomenon is known as “obesity paradox.”
- We have shown short- and long-term survival advantage in overweight and obese patients in comparison to normal weight individuals undergoing CABG.
- Our results confirming the existence of “obesity paradox” could contribute to optimization of risk assessment in patients presenting for cardiac surgery and thus improve the allocation of healthcare resources.

Even though the existence of “obesity paradox” is well documented, its underlying cause has not been clearly elucidated^[4,5]^.

Meanwhile, body weight as a potential risk factor is even more important in patients with CAD undergoing coronary artery bypass grafting (CABG). In this population, the BMI status impacts not only the course of the disease itself, but also the referral patterns and outcomes of surgery[7].

A substantial body of research has been published on the “obesity paradox” in patients undergoing CABG. Nevertheless the results and conclusions are conflicting. Some authors failed to note any protective effect of increased body weight in these patients^[8-11]^. Others demonstrated short-term reduction in mortality with increasing levels of obesity[12]. Of note, there are only few studies which showed better survival in obese patients at long-term follow up[13].

In summary, the impact of increased body weight on the outcomes of CABG remains unclear. It is however a clinically important issue, and the confirmation or rebuttal of the “obesity paradox” in the context of CABG could contribute to optimization of risk assessment in patients presenting for cardiac surgery and improve the allocation of resources.

Therefore, we performed a study to assess the relationship between BMI and mortality (short- and long-term) in patients following CABG in a single, large cardiothoracic center.

## Patients and methods

It is a retrospective cohort study which enrolled all consecutive patients who underwent isolated CABG at a tertiary center in Poland from January 2014 through September 2020. Procedural data were prospectively collected from our departmental registry of surgical procedures. The database is regularly validated. The local Institutional Review Board reviewed the study and did not categorize it as a medical experiment. The formal approval was waived (decision KNW/0022/KB/284/17 dated 12 December 2017). Based on above, the anonymized data were used for analysis and the individual informed consent was not obtained. Our work has been reported in line with the Strengthening the Reporting of Cohort Studies (STROCSS) criteria[14].

### Study population

All consecutive patients aged above 18 who underwent isolated CABG were enrolled. Patients operated on- and off-pump were included. We reviewed elective, urgent, emergency, and salvage surgeries. Patients with any additional concomitant surgical procedures were excluded.

Patients were divided into four groups according to BMI based on World Health Organization (WHO) guidelines: underweight (BMI < 20.0 kg/m^2^), normal weight (BMI = 20.0–24.9 kg/m^2^), overweight (BMI = 25–30 kg/m^2^), and obese (BMI > 30 kg/m^2^)[15].

The study population consisted of 6448 patients including 104 patients in the underweight group (1.6%), 1296 patients in the normal weight group (20.1%), 2946 patients in the overweight group (45.7%), and 2102 patients in the obese group (32,6%).

### Endpoints

The primary endpoint was long-term mortality. The secondary end-point was the rate of sternal wound infections.

### Statistical analysis

Summary statistics were calculated. Continuous data are described as the median, with the interquartile range in parentheses, while categorical data are shown as numbers with percentages. Differences between groups for normally distributed, continuous variables were determined using the one-way analysis of variance with Holm-Sidak *post-hoc* test. The Kruskal Wallis test was used for the analysis of non-normally distributed continuous variables with Dunn’s test for *post-hoc* comparison. The chi-squared test was used for the analysis of categorical variables. Bonferoni’s correction was used for *post-hoc* analysis. To assess the impact of variables on 30-day mortality the logistic regression was used. The uni- and multi-variable analysis of the impact of different variables on long-term mortality was assessed using the Cox regression. The variables were selected using the backward conditional method with variables with score statistics <0.1 included in the model. Significance was assessed at *P* < 0.05. Multiple imputation was not used to adjust for missing data. The number of valid entries for each variable and alterations in the sample size for specific analyses are described. Analyses were performed using IBM SPSS Version 22.0 and MedCalc Version 19.4.1.

## Results

### Patient’s characteristics

Patients’ baseline characteristics revealed significant differences between groups with regard to weight, NYHA class, ejection fraction (EF), and Euroscore II. Underweight patients had the highest Euroscore II of 2.4 (1.5–4.6), *P* < 0.001, and the lowest EF of 45% (38%–55%) compared to other study groups. There was no difference in the rate of diabetes between the BMI groups (*P* = 0.769). Complete baseline characteristics of the patients are presented in Table 1.

**Table 1:** Baseline characteristics

Variables	BMI<20 (*n* = 104)	BMI20-24.9 (*n* = 1296)	BMI25-30 (*n* = 2946)	BMI>30 (*n* = 2102)	*P*-value
Age	66(60–71)^a,b^	67(61–74)^a,b^	67(62–73)^a^	66(61–71)^b^	<0.001
Sex (female) (%)	37(36)^a^	351(27)^a^	640(22)^b^	578(28)^a^	<0.001
Weight (kg)	53(48–58)	67(62–72)	80(74–85)	94(87–102)	<0.001
Height (cm)	168(161–174)^a^	170(164–175)^a^	170(165–176)	170(163–175)^a^	<0.001
**BMI**	19.2 (18.4–19.8)	23.6 (22.5–24.4)	27.5 (26.3–28.7)	32.5 (31.1–34.6)	<0.001
CCS class(%)					0.030
I	20(20)^a,b^	272(21.8)^a^	633(22.1)^a^	384 (18.7)^b^	
II	41(41)^a^	587(47.1)^a^	1386(48.4)^a^	998 (48.7)^a^	
III	25(25)^a^	243(19.5)^a^	576(20.1)^a^	456 (22.3)^a^	
IV	14(14)^a,b^	143(11.5)^a^	269(9.4)^b^	211 (10.3)^a,b^	
ACS presence (%)	15(14.4)	174(13.4)	325(11)	247(11.8)	0.130
NYHA class (%)					<0.001
I	15(14.4)^b^	336(25.9)^a^	780(26.5)^a^	419(29.9)^b^	
II	72(69.2)^a^	866(66.8)^a^	1957(66.4)^a^	1444(68.7)^a^	
III	16(15.4)^b^	91(7)^a^	189(6.4)^a^	227(10.8)^b^	
IV	1(1)^a^	3(0.2)^a^	20(0.7)^a^	12(0.6)^a^	
Hypertension (%)	89(85.6)	1184(91.4)	2705(91.8)	1929(91.6)	0.153
Hyperlipidemia (%)	88(84.6)	1086(83.8)	2519(85.5)	1783(84.8)	0.554
Creatinine (mg/dl)	0.87	0.92	0.94	0.95	<0.001
	(0.75–1.03)^a^	(0.79–1.09)^a^	(0.81–1.1)^b^	(0.81–1.12)^b^	
eGFR (ml/min/1.73 m^2^)	82(64–90)	80(63–90)	80(65–90)	79(64–90)	0.456
Renal impairment(%)					0.237
Normal (GFR>85 ml/min)	49(47.1)	520(40.1)	1087(36.9)	783(37.3)	
Moderate (GFR 50–85 ml/min)	44(42.3)	635(49)	1549(52.6)	1095(52.1)	
Severe (GFR<50 ml/min)	9(8.7)	132(10.2)	290(9.8)	209(9.9)	
Dialysis	2(1.9)	9(0.7)	20(0.7)	15(0.7)	
EF (%)	45(38–55)	50(43–55)^a^	54(45–55)^b^	52(45–55)^a,b^	<0.001
Troponin (ng/ml)	0.020	0.015	0.015	0.015	0.040
	(0.011–0.057)^a^	(0.009–0.038)^a,b^	(0.009–0.033)^b^	(0.009–0.033)^a,b^	
MI in the past >3 months (%)	31(29.8)	347(26.8)	809(27.5)	584(27.8)	0.872
Acute MI					<0.001
NSTEMI(%)	37(35.6)^a^	381(29.4)^a^	776(26.3)^b^	524(24.9)^b^	
STEMI (%)	8(7.7)^a,b^	86(6.6)^a^	132(4.5)^b^	68(3.2)^c^	
Time from MI to surgery(%)					<0.001
<6 h	1(1.0)^a^	6(0.5)^a^	10(0.3)^a^	8(0.4)^a^	
1 day	0(0.0)^a^	21(1.6)^a^	47(1.6)^a^	24(1.1)^a^	
2–7 days	21(20.2)^a^	234(18.1)^a^	485(16.5)^a^	340(16.2)^a^	
2–3 weeks	13(12.5)^b^	74(5.7)^a^	137(4.7)^a,c^	81(3.9)^c^	
3 weeks to					
3 months	10(9.6)^a,b^	132(10.2)^a^	229(7.8)^b^	139(6.6)^b^	
Left main coronary artery stenosis (%)	35(33.7)^b^	304(23.5)^a^	702(23.8)^a^	466(22.2)^a^	0.042
Mechanical ventilation (%)	1(1.0)^b^	0(0.0)^a^	3(0.1)^a^	3(0.1)^a,b^	0.035
Cardiogenic shock (%)	1(1.0)	5(0.4)	5(0.2)	5(0.2)	0.276
Inotropes (%)	1(1.0)	3(0.2)	9(0.3)	6(0.3)	0.622
Preoperative IABP (%)	3(2.9)	32(2.5)	60(2.0)	30(1.4)	0.142
COPD (%)	7(6.7)	75(5.8)	158(5.4)	142(6.8)	0.218
Permanent pacemaker (%)	1(1.0)	24(1.9)	61(2.1)	49(2.3)	0.658
Extracardiac Arteriopathy (%)	42(40.4)^b^	350(27)^a^	696(23.6)^c^	495(23.5)^c^	<0.001
PVD (%)	27(26.0)^b^	196(15.1)^a^	397(13.5)^a,c^	248(11.8)^c^	<0.001
Cerebrovascular disease (%)	21(20.2)^b^	185(14.3)^a,b^	340(11.5)^c^	280(13.3)^a,c^	0.006
DM (%)	37(35.6)	449(34.7)	1009(34.2)	772(36.8)	0.769
EuroSCOREII	2.4(1.5–4.6)	1.9(1.2–3.1)	1.5(1.0–2.5)	1.4(0.9–2.3)	<0.001
Smoking					0.192
Actual (%)	19(18.3)	252(19.4)	470(16)	349(16.6)	
In the past (%)	58(55.8)	744(57.4)	1777(60.3)	1257(59.8)	
Never smoked (%)	27(26.0)	300(23.1)	699(23.7)	496(23.6)	

Data presented as median with quartiles or n with %. Superscripts denote groups with no statistically significant difference on *post-hoc* test.

**Abbreviations**: CCS, Canadian Cardiovascular Society; ACS, acute coronary syndrome; NYHA, New York Heart Association; eGFR, estimated glomerular filtration rate; EF, ejection fraction; MI, myocardial infarction; STEMI, ST-elevation myocardial infarction; NSTEMI, non ST-elevation myocardial infarction; LM, left main stem; COPD, chronic obstructive pulmonary disease; AF, atrial fibrillation; PVD, peripheral vascular disease; DM, diabetes mellitus.

### Operative details

Operative data revealed the same number of grafts constructed in each group. However, the left internal thoracic artery was used less often in the underweight patients (87.5% vs 93.9%; *P* = 0.002). The overweight and obese patients less frequently underwent off-pump surgery than normal weight patients (15.7% and 16.5% vs 19.3%, respectively). The full set of perioperative data is presented in Table 2.

**Table 2:** Operative data

Variables	BMI <20 (*n* = 104)	BMI 20 to 25 (*n* = 1296)	BMI 25 to 30 (*n* = 2946)	BMI>30 (*n* = 2102)	*P*-value
Off pump	23(22.1)^a,b^	250(19.3)^a^	462(15.7)^b^	346(16.5)^b^	0.014
CPB (min)	59(41.5–73.5)	55(42–69)	54(42–68)	54(42–68)	0.558
Xclamp	33(23–41)	30(23–38)	31(23–39)	30(23–39)	0.445
No of grafts	2(2–3)^a,b^	2(2–3)^a,b^	2(2–3)^a^	2(2–3)^b^	0.014
LIMA use(%)	91(87.5)^b^	1197(92.4)^a,b^	2786(94.6)^c^	2102(93.9)^a,c^	0.002

**Abbreviations**: CPB, cardiopulmonary bypass; LIMA, left internal mammary artery.

Data presented as median with quartiles or n with %. Superscripts denote groups with no statistically significant difference on *post-hoc* test.

### Postoperative outcomes

Overall 30-day mortality was 2.5% in the whole study population while 9.6%, 3.1%, 1.9%, and 2.6% in respective study groups (*P* < 0.001). Conversely, the sternal wound infection rate was the highest in the obese group and it increased with increasing BMI (underweight 1.0% vs normal weight 1.2% vs overweight 1.4% vs obese 2.9%; *P* < 0.001) (Table 3).

**Table 3:** Postoperative outcomes

Variables	BMI < 20 (*n* = 104)	BMI 20–24.9 (*n* = 1296)	BMI 25–30 (*n* = 2946)	BMI > 30 (*n* = 2102)	*P*-value
30-day mortality (%)	10(9.6)^a^	40(3.1)^b^	57(1.9)^c^	51(2.6)^b,c^	<0.001
Sternal wound infection (%)	1(1.0)^a,b^	16(1.2)^a^	42(1.4)^a^	62(2.9)^b^	<0.001
Total postoperative chest drain output (ml)	605(450–870)^a,b^	600(459–825)^a^	600(440–780)^a^	570(420–740)^b^	<0.001
Chest reexploration for postoperative bleeding (%)	14(13.5)^a^	70(5.4)^b^	136(4.6)^b^	69(3.3)^c^	<0.001
CVA (%)	0(0)	28(2.2)	40(1.4)	23(1.4)	0.179
Psychosis (%)	17(16.3)	116(9.0)	181(6.1)	110(6.7)	<0.001
Postoperative troponin (ng/ml)*	0.320(0.187–0.600)^a,b^	0.279(0.177–0.492)^b^	0.250(0.160–0.440)^a^	0.240(0.150–0.396)	<0.001
IABP(%)	11(10.6)	98(7.6)	182(6.2)	123(5.9)	0.065
Respiratory complications (%)	8(7.7)	41(3.2)	95(3.2)	54(3.3)	0.124
IPPV (h)	12.5(10.0–17.7)^a^	11.8(9.1–15.3)^a^	11.3(8.9–14.1)^b^	10.9(8.8–14.2)^b^	<0.001
Hospitalization (days)	7(7–9)	7(7–9)	7(7–8)	7(7–9)	0.065

^*^ Postoperative troponin value was regularly measured on postoperative day 1. Data presented as median with quartiles or *n* with %. Superscripts denote groups with no statistically significant difference on *post-hoc* test.

**Abbreviations**: CVA, cerebrovascular accident; IABP, intra-aortic balloon pump; IPPV, intermittent positive pressure ventilation; NPWT, negative pressure wound therapy.

The underweight group had the lowest sternal wound infection rate in spite of the fact that these patients had the highest postoperative chest tube drainage volume (underweight 605 ml vs normal weight 600 ml vs overweight 600 ml vs obese 570 ml; *P* < 0.001), and the highest rate of chest reexploration for bleeding (underweight 13.5% vs normal weight 5.4 % vs overweight 4.6 % vs obese 3.3 %; *P* < 0.001). Underweight patients spent more time on ventilator, more frequently had psychosis and elevated troponin levels (for all *P* < 0.001). Complete postoperative data are presented in Table 3.

At 30-days after surgery the adjusted mortality risk was significantly higher in the underweight group, OR 2.904, 95% CI 1.286–6.556 (*P* = 0.010), whereas the overweight patients had survival benefit over the normal weight group, OR 0.652, 95% CI 0.425–0.998 (*P* = 0.049).

**Figure 1:**
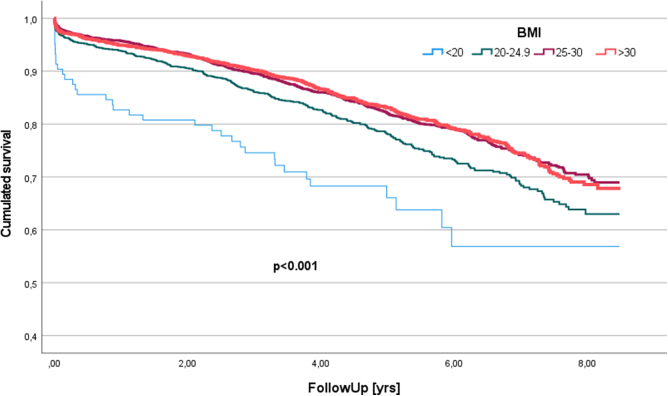
Kaplan–Meier curves indicating survival from the date of surgery in respective body mass index categories (*P* < 0.001).

The 5-year survival rate were 66 ± 5.1%, 78 ± 1.3%, 82 ± 0.8%, and 83 ± 0.9% in underweight, normal weight, overweight, and obese groups, respectively (*P* < 0.001) (Fig. 1).

Univariable analysis showed survival benefit in all patients with increased weight in comparison to normal weight group; with HR 0.776, 95% CI 0.675–0.891, *P* < 0.001 in the overweight, and HR 0.767, 95% CI 0.661–0.890, *P* < 0.001 in the obese patients.

After adjustment for significant covariables (Table 4), multivariable analysis revealed better survival in overweight and obese patients in comparison to normal weight group (HR 0.836, 95% CI 0.727–0.961, *P* = 0.012, HR 0.854, 95% CI 0.734–0.994, *P* = 0.042, respectively).

**Table 4:** Multivariate cox analysis

Variable	Hazard ratio	95% CI	*P*-value
BMI			0.004
Underweight (BMI < 20)*	1.375	0.964–1.960	0.079
Overweight (BMI 25–30)*	0.836	0.727–0.961	0.012
Obese (BMI > 30)*	0.854	0.734–0.994	0.042
Age at admission	1.035	1.028–1.043	<0.001
NYHA			0.005
Class II†	1.038	0.911–1.184	0.572
Class III†	1.309	1.079–1.588	0.006
Class IV†	2.008	1.096–3.680	0.024
GFR	0.987	0.984–0.990	<0.001
Troponin at admission	1.261	1.155–1.376	<0.001
History of MI	1.231	1.097–1.382	<0.001
Moderate MR	1.190	1.021–1.388	0.026
Mechanical ventilation preop	2.908	1.035–8.170	0.043
Inotropes preop	2.043	1.020–4.096	0.044
COPD preop	1.264	1.037–1.539	0.020
AF preop	1.275	1.066–1.524	0.008
Pacing preop	1.497	1.122–1.997	0.006
PVD	1.553	1.354–1.781	<0.001
Cerebral vessel disease	1.300	1.128–1.498	<0.001
LVEF	0.975	0.970–0.981	<0.001

^*^ vs normal weight BMI^†^ vs NYHA I Hazard ratios for factors with significant effect on mortality. Each line represents the effect of that factor, adjusted for all other factors in the model.

BMI, body mass index; NYHA, New York Heart Association; GFR, glomerular filtration rate; MI, myocardial infarction; MR, mitral regurgitation; AF, atrial fibrillation; COPD, chronic obstructive pulmonary disease; PVD, peripheral vascular disease; LVEF, left ventricular ejection fraction.

## Discussion

In this study, we have demonstrated that overweight patients undergoing CABG have lower in-hospital mortality and both overweight and obese patients have long-term survival benefit in comparison to those with normal BMI. Our findings support the notion of “obesity paradox” being relevant to patients undergoing surgical coronary revascularization. The obesity paradox is referring to overweight and obese patients who on one hand have greater risk of acquiring CAD, but on the other hand, once the disease has been diagnosed they seem to have lower mortality than normal-weight individuals. While the paradox is well documented in the general population with CAD, it is more controversial in the subgroup of patients undergoing CABG.

There is a group of researchers who failed to identify the “obesity paradox” in these patients.

Prabhakar *et al* analyzed the Society of Thoracic Surgeons database (large population of 559 004 patients who underwent isolated CABG) and found higher in-hospital mortality in moderately (BMI 35–39.9 kg/m^2^) and severely (BMI > 40 kg/m^2^) obese patients in comparison to patients with a BMI of 18.5–34.9 kg/m^2 [8]^.

Gurm *et al* reviewed the data from the Bypass Angioplasty Revascularization Investigation (BARI) registry of 1526 patients who had CABG. There was no difference observed in major in-hospital events (death, myocardial infarction, stroke, and coma) according to BMI. At long-term BMI was positively associated with the risk of 5-year mortality, especially cardiac mortality[9].

Van Straten *et al* investigated the impact of BMI on a large group of 10 268 patients who underwent CABG. They observed no association between moderate obesity and early and late mortality in this population[10].

Benedetto *et al* used propensity score-matched analysis to examine the existence of “obesity paradox” but concluded that obesity did not confer any protective effect on early and late mortality in patients undergoing CABG[11].

There is nevertheless a body of evidence confirming better outcomes of CABG in overweight and moderately obese patients. Most of these data relate to short-term postoperative follow-up and suggests lower 30-day or in-hospital mortality in this population comparing to normal-weight individuals[12]. This phenomenon could be explained by the protective effect of adipose tissue in the early period of post-operative stress. Subcutaneous fat accumulation has a positive impact on cardiac metabolism. It is less infiltrated by pro-inflammatory M1 macrophages but has the ability to increased accumulation of anti-inflammatory and insulin-sensitizing M2 macrophages. Adipose tissue has been also shown to increase the expression of adipokines and anti-inflammatory cytokines which reduce the oxidative stress^[16,17]^.

Data on the “obesity paradox” in the CABG patients extending beyond the early follow-up period is more scarce. Johnson *et al* analyzed 78 762 patients who had undergone first time CABG or combined CABG/aortic valve replacement (AVR). The follow-up time was 7.8 ± 4 years. He found a reverse J-curve relationship between BMI and mortality, with underweight and morbidly obese patients having significantly higher risk of death. There was a survival benefit in the overweight group and no difference in the obese group, compared to normal weight[13]. These results are similar to our findings with regard to worse outcome in patients with weight extremes.

The novelty of our findings however is that we have shown the long-term survival advantage not only in the overweight but also in the obese patients. In other words, we have demonstrated that patients with excess weight undergoing CABG have similar long-term mortality risk profile as those with CAD in the general population[6]. In our study group, the survival benefit persists despite the sternal wound infection rate rising with increasing body weight.

The cause of the “obesity paradox” at long-term follow up after CABG is unclear. As the stress associated with surgery abates over time, the protective effects of adipose tissue potentially beneficial in the early period, become less important. Some authors point out that the problems with explaining the “obesity paradox” may in part be related to the shortcomings of BMI as a measure of obesity. BMI does not distinguish between lean mass and fat mass and consequently people with higher BMI may not necessarily have higher fat mass. Indeed increased lean mass in obese patients has been associated with better long-term outcomes in patients with CAD^[5,18]^. Second, obese patients in comparison to normal weight individuals are more likely to receive aggressive treatment for CAD and be involved in secondary prevention programs such as healthy diet, physical activity, and social media support groups. It has also been suggested that improved outcomes in obese patients with CAD may be linked to higher counts of circulating progenitor cells (CPC), which are a measure of intrinsic regenerative capacity. In a study by Mehta *et al* obesity was associated with 8–12% higher CPC counts and 30% lower risk of adverse outcomes[19].

We have found that the underweight patients have the highest mortality both short- and long term. Indeed, the underweight had the worst baseline health status in comparison with the remaining study population (more frequent myocardial infarction, higher preoperative troponin value, lower EF, and higher EuroScore). Moreover, in the multivariable analysis, adjusting for EuroScore II factors like: age, glomerular filtration rate, acute myocardial infarction, the mortality risk was still the highest in the underweight patients (Table 4). Our findings correspond to the results published by other authors, who identified low BMI as an independent risk factor of increased morbidity and mortality in patients undergoing CABG^[10,12,13]^.

### Study limitations

It was a retrospective analysis which could introduce unknown bias. It is possible that obese who were considered as high operative risk patients were not referred to surgery. We did not exclude patients with acute weight gain or loss, severe chronic diseases or cardiac cachexia which could have influenced our results. Moreover the quantity of patients differs significantly between BMI groups. Underweight (104 patients, 1.6%) group is relatively small to give a strongly meaningful data but it determines clear trends in mortality and sternal wound infections. Nonetheless, it reaffirms the long held belief that grossly underweight patients have an increased risk of dying. On the other hand, we included patients below BMI 20 in the underweight group. This was done to increase the number of patients in the underweight group and to improve statistical inference. It however means that not only cachectic patients represented this population. Future larger prospective studies are warranted to further investigate the issue of “obesity paradox” in patients undergoing CABG.

## Conclusions

We conclude that increased weight is associated with better long-term survival in patients after CABG. This effect is present both in overweight (BMI 25–30 kg/m^2^) and obese (BMI >30 kg/m^2^) patients. It persists despite the sternal wound infection rate rising with increasing body weight.

## Data Availability

All datasets generated and analyzed during this study are available upon reasonable request.

## Research Registration Unique Identifying Number (UIN)

The research was retrospectively registered at clinicaltrials.gov and a following ID was assigned: NCT06614023.
